# Poly (I:C), an agonist of toll-like receptor-3, inhibits replication of the Chikungunya virus in BEAS-2B cells

**DOI:** 10.1186/1743-422X-9-114

**Published:** 2012-06-14

**Authors:** Yong-Gang Li, Uamporn Siripanyaphinyo, Uranan Tumkosit, Nitchakarn Noranate, Atchareeya A-nuegoonpipat, Yang Pan, Masanori Kameoka, Takeshi Kurosu, Kazuyoshi Ikuta, Naokazu Takeda, Surapee Anantapreecha

**Affiliations:** 1 Department of Virology, Research Institute for Microbial Diseases, Osaka University, Osaka, 565-0871, Japan; 2 Section of Viral Infections, Thailand-Japan Research Collaboration Center on Emerging and Re-emerging Infections, Nonthaburi, 11000, Thailand; 3 National Institute of Health, Department of Medical Sciences, Ministry of Public Health, Nonthaburi, 11000, Thailand

**Keywords:** Chikungunya virus, Poly (I:C), BEAS-2B cells, TLR3

## Abstract

**Background:**

Double-stranded RNA (dsRNA) and its mimic, polyinosinic acid: polycytidylic acid [Poly (I:C)], are recognized by toll-like receptor 3 (TLR3) and induce interferon (IFN)-β in many cell types. Poly (I:C) is the most potent IFN inducer. In *in vivo* mouse studies, intraperitoneal injection of Poly (I:C) elicited IFN-α/β production and natural killer (NK) cells activation. The TLR3 pathway is suggested to contribute to innate immune responses against many viruses, including influenza virus, respiratory syncytial virus, herpes simplex virus 2, and murine cytomegalovirus. In Chikungunya virus (CHIKV) infection, the viruses are cleared within 7–10 days postinfection before adaptive immune responses emerge. The innate immune response is important for CHIKV clearance.

**Results:**

The effects of Poly (I:C) on the replication of CHIKV in human bronchial epithelial cells, BEAS-2B, were studied. Poly (I:C) suppressed cytopathic effects (CPE) induced by CHIKV infection in BEAS-2B cells in the presence of Poly (I:C) and inhibited the replication of CHIKV in the cells. The virus titers of Poly (I:C)-treated cells were much lower compared with those of untreated cells. CHIKV infection and Poly (I:C) treatment of BEAS-2B cells induced the production of IFN-β and increased the expression of anti-viral genes, including IFN-α, IFN-β, MxA, and OAS. Both Poly (I:C) and CHIKV infection upregulate the expression of TLR3 in BEAS-2B cells.

**Conclusions:**

CHIKV is sensitive to innate immune response induced by Poly (I:C). The inhibition of CHIKV replication by Poly (I:C) may be through the induction of TLR3, which triggers the production of IFNs and other anti-viral genes. The innate immune response is important to clear CHIKV in infected cells.

## Introduction

Chikungunya virus (CHIKV), the causative agent for Chikungunya fever, was first described in 1952 during an epidemic in Tanzania, East Africa [1,2]. CHIKV is a positive-sense single-strand RNA virus belonging to the genus *Alphavirus* of the family *Togaviridae*, and it is maintained in two distinct transmission cycles, a sylvatic cycle and a human-mosquito-human cycle. The scale of epidemics of the former is smaller and is mainly confined within African countries, involving primates such as monkeys and forest-dwelling Aedes mosquitoes [3]. CHIKV is mainly transmitted by *Aedes aegypti* and *Aedes albopictus.* CHIKV epidemics have often been characterized by long interepidemic (more than 10 years) periods in many parts of Southern and Southeast Asia [4-7]. During the past 8 years, major outbreaks have occurred among islands in the Indian Ocean, with Reunion Island being one of the most severely hit islands. One-third of its population were infected, and more than 240 people died [8-12]. The symptoms of Chikungunya generally start 4–7 d after the bite. Acute infection lasts 1–10 days and is characterized by a painful polyarthralgia, high fever, asthenia, headache, vomiting, rash, and myalgia [13,14]. CHIKV infection has affected as many as 3–4 million people in the Indian Ocean zone, and it spread to Europe in 2005–2007. This disease has recently received considerable attention in Thailand [15-19].

CHIKV transmission is rapid and extensive; however, humans are not defenseless, and in fact, CHIKV is efficiently cleared within 4–7 days after infection *in vivo*[20-22]. As a typical adaptive immune response, such as CHIKV-specific B-cell and T-cell activation, requires at least 1 week for development, the innate immune system seems to control CHIKV infection [23]. CHIKV is known to infect many different cell types, including fibroblasts and epithelial and endothelial cells *in vitro*[24] and fibroblast cells *in vivo*[25]; however, epithelial cells are armed with various mechanisms that are able to sense viral components and initiate intracellular signal transduction to respond rapidly to viral infections [26]. Polyinosinic: polycytidylic acid [Poly (I:C)], a synthetic double-stranded RNA (dsRNA) analog, is an immunostimulant that acts as the most potent interferon (IFN) inducer [27]. In *in vivo* mouse studies, intraperitoneal injection of Poly (I:C) elicited IFN-α/β production and natural killer (NK) cells activation [28,29]. Poly (I:C) is known to interact with toll-like receptor 3 (TLR3), which is expressed in the membrane of B-cells, macrophages, and dendritic cells.

TLRs are a member of the family of host innate immune receptors, and they are essential for detecting pathogen-associated molecular patterns. TLRs are transmembrane signaling proteins designed to specifically recognize various proteins, carbohydrates, lipids, and nucleic acids of invading microorganisms. When a TLR is activated, it triggers immune and inflammatory responses to infectious agents [30]. The TLR3 pathway contributes to an innate immune response against many viruses, including influenza virus [31], respiratory syncytial virus [32], herpes simplex virus 2 [33], and murine cytomegalovirus [34]. The detection of viral dsRNA and Poly(I:C) in the cytosol is mediated through the helicase family members retinoic-acid-inducible gene I (RIG-I) and melanoma-differentiation-associated gene 5 (MDA-5), thus allowing the host to sense directly an intracellular viral infection in a TLR3-independent way [35,36]. *In vitro* studies have shown that RIG-I and MDA-5 are both capable of responding to Poly(I:C) and RNA viruses [37].

In this study, Poly (I:C) was used to examine the innate immune response *in vitro*. We found that Poly (I:C) suppressed the cytopathic effect (CPE) induced by CHIKV infection and inhibited the replication of CHIKV in human bronchial epithelial-derived cells, BEAS-2B, by inducing the expression of IFNs and interferon-inducible intracellular antiviral factor genes, including OAS and MxA. Based on our results, we concluded that the CHIKV was sensitive to IFNs and that the innate immune response plays an important role in the clearance of CHIKV.

## Results

### Poly (I:C) suppressed CPE induced by CHIKV infection

**Figure 1:**
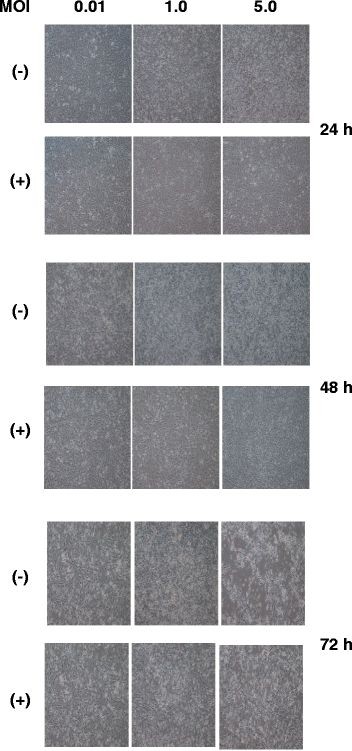
**Effect of Poly (I:C) treatment on CPE.** BEAS-2B cells were treated with 4 μg/ml of Poly (I:C) **(+)** or mock-treated **(−)** for 1 h, and then infected with CHIKV at MOI 0.01, 1, and 5. At 24, 48, and 72 h p.i., CPE was observed under a microscope.

BEAS-2B cells were seeded in 6-well plates (1x10^6^ cells/well) one day before Poly (I:C) treatment. One hour before infection at multiplicity of infection (MOI) 0.01, 1, or 5, the cells were pre-treated with 4 μg/ml of Poly (I:C) or left untreated. After adsorption, the cells were maintained in the medium with or without Poly (I: C) (4 μg/ml). The CPE was observed at 24, 48, and 72 h postinfection (p.i.) under a microscope. No CPE was found in Poly (I:C)-treated cells at 24 or 48 h p.i., even when MOI 5 was used. Although CPE was found in the Poly (I:C)-treated cells at 72 h p.i., it was less significant compared with that of untreated cells (Figure 1), demonstrating that Poly (I:C) treatment appeared to decrease CPE induced by CHIKV infection. Because the protection of CPE was decreased at 72 h p.i., we conclude that the protection is important in the early phase of infection.

### Poly (I:C) inhibited replication of CHIKV in BEAS-2B cells

**Figure 2:**
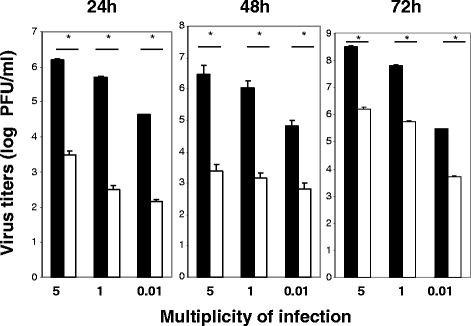
**Effect of Poly (I:C) treatment on CHIKV growth.** BEAS-2B cells were treated with 4 μg/ml of Poly (I:C) **(□)** or mock-treated **(■)** for 1 h, and then infected with CHIKV at MOI 0.01, 1, and 5. At 24, 48, and 72 h p.i., the virus titer in the supernatant was measured by a plaque assay. *P < 0.01 by Student’s unpaired *t*-test.

Since the Poly(I:C) decreased CPE in BEAS-2B cells induced by CHIKV infection, we supposed that Poly(I:C) may inhibit the replication of CHIKV. To clarify the effect of Poly (I:C) treatment, we measured the virus titers produced by Poly (I:C)-treated and mock-treated cells by plaque assay (Figure 2). The supernatant was collected at 24, 48, and 72 h p.i. at each MOI. The virus titers from mock-treated cells were 1.5x10^6^, 5.5x10^5^, and 4.5x10^3^ pfu/ml at MOI 0.01; 4.3x10^6^, 1x10^6^, and 5x10^4^ pfu/ml at MOI 1; 3.5x10^8^, 6.9x10^7^, and 3x10^5^ pfu/ml at MOI 5 at 24, 48, and 72 h p.i., respectively. The virus titers of the supernatant from Poly (I:C)-treated cells were 2.5x10^2^, 1.5x10^2^, and 1x10^2^ pfu/ml at MOI 0.01; 2.5x10^3^, 2x10^3^, and 6.3x10^2^ pfu/ml at MOI 1; 2x10^6^, 6x10^5^, and 5.5x10^3^ pfu/ml at MOI 5 at 24, 48, and 72 h p.i., respectively, indicating that Poly (I:C) treatment significantly lowers the virus titers. With either Poly (I:C) treatment or non-treatment, the virus titers showed a high peak at 24 h p.i. in the infections with the same MOIs and a trend to decrease at 48 and 72 h p.i. These results indicated that Poly (I:C) inhibited the replication of CHIKV in BEAS-2B cells. This is probably because IFN-β induced by Poly (I:C) treatment plays a role, as described previously [31].

### Induction of IFN-β and stimulation of TLR3 expression in BEAS-2B cells by poly (I:C) treatment or CHIKV infection

**Figure 3:**
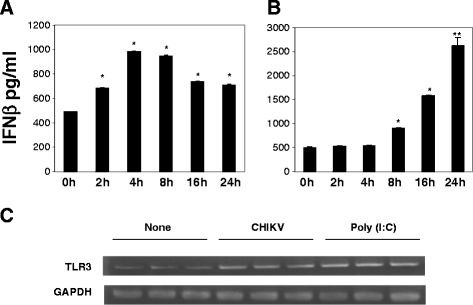
**Induction of IFN-β and expression of TLR3 in BEAS-2B cells treated with Poly (I:C) or infected with CHIKV.** (**A**) BEAS-2B cells were incubated in the presence of 4 μg/ml of Poly (I:C), and IFN-β secreted in the medium was measured by an ELISA. The samples were collected at 0, 2, 4, 8, 16, and 24 p.i. (**B**) BEAS-2B cells were infected with CHIKV at MOI 0.8. IFN-β in the medium was measured by an ELISA at 0, 2, 4, 8, 16, and 24 h p.i.. * P < 0.05: ** P < 0.01 relative to the 0 h time point. (**C**) Expression of TLR3 in BEAS-2B cells was detected by RT-PCR. The cells were incubated with 4 μg/ml of Poly (I:C) or infected with CHIKV at MOI 0.8. Total RNA was extracted from the cells at 24 h p.i., and TLR3 mRNA was amplified by RT-PCR. The products were analyzed by agarose gel electrophoresis. A representative result of the experiment performed in triplicate is shown.

Poly (I:C) is a strong IFN inducer. The effects of Poly (I:C) treatment on CHIKV infection in BEAS-2B cells may be due to the production of IFNs. To elucidate the level of IFN-β, we treated BEAS-2B cells with 4 μg/ml of Poly (I:C) and measured the amount of IFN-β by ELISA. The concentration of IFN-β in the supernatant at 0, 2, 4, 8, 16, and 24 h p.i. was 490.60, 681.69, 984.61, 947.82, 736.91, and 710.54 pg/ml, respectively, indicating that Poly (I:C) treatment induced the secretion of IFN-β. The IFN-β level reached a peak at 4 h during the treatment (Figure 3A). The IFN-β was also induced by CHIKV infection (MOI 0.8) and reached a peak at 24 hours p.i. The concentration at 0, 2, 4, 8, 16, and 24 h p.i. was 501.54, 526.67, 547.23, 907.43, 1585.95, and 2614.92 pg/ml, respectively, (Figure 3B). TLR3 was known as a receptor for dsRNA [38,39], and upon recognition of dsRNA, TLR3 transmits signals that activate the transcript factors IFR-3, NF-ҚB, and AP-1, leading to the induction of type I IFN [40][41]. The level of TLR3 expression examined by PCR is shown in Figure 3C. The expression of TLR3 mRNA was upregulated by both Poly (I:C) treatment and CHIKV infection after 24 hours. The induction of IFN-β may be triggered through the upregulated expression of TLR3.

### Induction of IFN-α, IFN-β, MxA, and OAS genes

**Figure 4:**
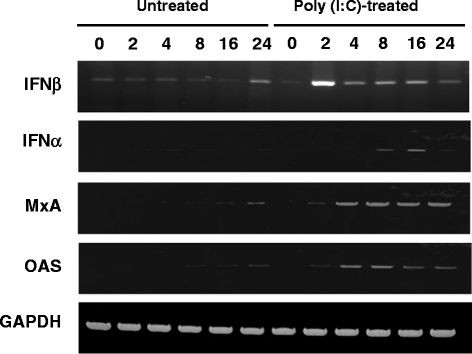
**Expression of anti-viral genes after Poly (I:C) treatment.** BEAS-2B cells were treated or not treated with Poly (I:C), and a total RNA was extracted from the cells at 0, 2, 4, 8, 16, and 24 h p.i. The IFN-β, IFN-α, MxA, and OAS genes were amplified by RT-PCR, and the products were analyzed by agarose gel electrophoresis.

One unique feature of TLR3 is to trigger the induction of the type I IFNs (IFN-α/β). In addition TLR3 is known to induce the expression of interferon-inducible intracellular antiviral factors including OAS and MxA [42,43]. We examined the expression of mRNA of these genes using RT-PCR. As shown in Figure 4, Poly (I:C) treatment stimulated the induction of IFN-β mRNA, and a significant upregulation was observed at 2 h post treatment. The expression level was still apparent, albeit at a lower level, at 4 and 8 h post treatment. A significant upregulation of IFN-α was observed at 16 h poststimulation. Exposure of BEAS-2B cells to Poly (I:C) induced time-dependent expression of MxA and OAS mRNA; however, unlike IFN-α/β, the levels of these two transcripts remained elevated 4, 8, 16, and 24 h poststimulation (Figure 4.) These results indicated that Poly (I:C) induced the anti-viral genes that may contribute to the inhibition of CHIKV replication.

## Discussion

The innate immune response is the first barrier against the viruses [44], initiated within hours after the viruses bind to the receptor, and it plays a central role in the detection of invading pathogens. The innate immune system responds through activating inflammatory and antiviral defense mechanisms against the infectious agents [45]. Innate immunity involves the induction of many factors, including IFNs-α/β, which induce a range of antiviral processes. In infected cells, it is believed that the proximal inducer of IFNs-α/β is intracellular dsRNA generated as an intermediate during viral replication [38].

During virus replication, not only dsRNA but also single-stranded RNA (ssRNA) molecules are recognized as intermediate by TLRs expressed in dendritic cells, natural killer cells, and macrophages, as well as in epithelium [46]. The dsRNA triggers a series of events culminating in the activation of PKR and other kinases. Phosphorylation of the substrates of these enzymes results in the translocation of transcription factors, NF-ҚB and IRF-3, from the cytoplasm to the nucleus, where they bind to the IFN-β promoter to form a transcription complex that ultimately drives IFN-β production [47,48][49,50]. Several *in vitro* studies have demonstrated that Poly (I:C), a TLR3 agonist, induces antiviral responses through the induction of IFN-β [51].

In the present study, we demonstrated that following Poly (I:C) treatment, BEAS-2B cells produced antimicrobial factors IFN-β, OAS, and MxA, which may constitute a highly specific and potent barrier against CHIKV infection. Poly (I:C) is known to markedly upregulate the IFN-β mRNA level in a dose-dependent manner in mouse osteoblastic MC3T3-E1 cells [52]. Similarly, trophoblast cells are known to express and secrete antiviral factors, such as OAS, MxA, and APOBEC3G, by Poly (I:C) [53]. Poly (I:C) treatment also inhibited the multiplication of xenotropic baboon type C endogenous retrovirus M7 in chronically infected human AV3-M7 cells [54] and human immunodeficiency virus amplification in dendritic cells via type I IFN-mediated activation of APOBEC3G [55].

Based on the results of the present study, we conclude that both Poly (I:C) and CHIKV infection enhanced the expression of TLR3. The stimulation of TLR3 by dsRNA transduces signals to activate the transcription factors NF-ҚB and IRF/interferon-sensitive response element (ISRE) via myeloid differentiation factor 88 (MyD88)-independent signaling pathways, which involve a distinct adaptor molecule, namely the Toll-interleukin (IL)-1 receptor (TIR) domain containing adaptor-inducing IFN-β (TRIF), also called the TIR domain containing adaptor molecule 1 (TICAM-1) [56,40]. This molecule elicits an antiviral response, especially through the production of IFNs-α/β [57]. Therefore, IFNs could contribute to decrease the CPE and inhibit the replication of CHIKV through TLR3 stimulation. Similar phenomena were reported in influenza virus in BEAS-2B cells. Both Poly (I:C) and influenza virus infection induced IFN- β [31]. The replication of CHIKV is controlled by IFNs-α/β [24], which is critically dependent on the action of nonhematopoietic cells through the induction of one or more IFN-stimulated genes (ISGs) [58]. Therefore, induction of IFNs and antiviral genes observed in this study could contribute to the Poly (I:C)-mediated suppression of CPE and inhibition of the replication of CHIKV in BEAS-2B cells.

Poly (I:C) was widely used as an adjuvant for vaccine research. Poly (I:C)-combined intranasal vaccine protected mice against influenza virus infection, including that due to highly pathogenic H5N1 [59-61]. Synthetic dsRNA is adjuvant for the induction of T helper 1 and humoral immune response to human papillomavirus in rhesus macaques [62]. Therefore, Poly (I:C) could be an adjuvant for CHIKV vaccine, which can increase the immune response in humans to clear the CHIKV.

## Conclusions

CHIKV is sensitive to innate immune response induced by Poly (I:C). Poly (I:C) decreased CPE and inhibited the CHIKV replication in BEAS-2B cells. The Poly (I:C) inhibition of CHIKV replication may be through the induction of TLR3, which triggers the production of IFNs and other anti-viral genes, such as MxA and OAS. The innate immune response is important to clear CHIKV in infected cells.

## Materials and methods

### Viruses, cells, and reagents

Chikungunya viruses (Ross Strain) were propagated in Vero-E6 (Vero) cells. The virus titer was measured by a plaque assay. BEAS-2B, a SV-40-transformed airway bronchial epithelial cell line, was purchased from American Type Culture Collection (Manassas, VA). Cells were maintained in RPMI-1640 supplemented with 10% FCS. All experiments were performed in a biosafety level 3 containment laboratory. Poly (I:C) was purchased from Sigma-Aldrich (St. Louis, MO).

### RT-PCR

Total RNA was extracted from the cells by using TRIzoL (Invitrogen, Carlsbad, CA) according to the manufacturer’s protocol. RT was performed using 3.5 μg of total RNA. PCR was performed using an INF-α forward primer (5’-TTTCTCCTGCCTGAAGGACAG-3’) and an INF-α reverse primer (5’-TCTCATGATTTCTGCTCTGACA-3’), a IFN-β forward primer (5’-CTGTGGCAATTGAATGGGAGGC-3’) and a IFN-β reverse primer (5’-CAGGCACAGTGACTGTCCTCCTT-3’), a MxA forward primer (5’-CATACTGCGAGGAGATCCTCCTT-3’) and a MxA reverse primer (5’-AGCATCCGAAATCTCAATCTCGTA-3’), a OAS forward primer (5’-AGAATGTCAGACACTGATCGACGA-3’) and a OAS reverse primer (5’-TGTTCCCAGGCATACACCGTA-3’), a TLR3 forward primer (5’-AAATTGGGCAAGAACTCACAGG-3’) and a TLR3 reverse primer (5’-GTGTTTCCAGAGCCGTGCTAA-3’), and a GAPDH forward primer (5’-CACCACCAACTGCTTAGCAC-3’) and a GAPDH reverse primer (5’-CCCTGTTGCTGTAGCCAAAT-3’). Amplification products were resolved on 1.5% agarose gel containing ethidium bromide.

### Plaque assay

Vero cells were seeded at 2.5x10^5^ cells per well in 24-wells plates, incubated at 37°C overnight, and washed once with phosphate buffered saline (PBS). Ten-fold serial dilutions of the virus mixture were prepared in Hanks buffer (Sigma-Aldrich), and then 0.1 ml of the mixture was inoculated into each well and incubated for 1 h at 37°C, during which we agitated the plate every 15 minutes. After adsorption for 1 h, the plate was washed with PBS three times, and 1 ml of DMEM containing 2% carboxymethyl cellulose (W/V) (Sigma-Aldrich) and 5% FBS was layered onto the cells. The plates were incubated in a humidified incubator at 37°C with 5% CO_2_ for 3 days. The overlay was removed and washed with PBS. Plaques were visualized by staining the monolayer with 1 ml 0.5% crystal violet containing 10% formaldehyde (Sigma-Aldrich) for 2 h at room temperature. The virus plaques were counted after thorough washing with tap water.

### Cytokine measurements

The concentration of human IFN-β in cell culture supernatants was determined by using DuoSet Elisa kits (R&D Systems, Minneapolis, MN).

## Competing interests

The authors declare that they have no competing interests.

## Authors’ contributions

YG. Li and SA conceived and designed the experiment. US, UT, NN, AA, YP. MK, KT. Performed the experiments. KI, NT. YG. LI and SA analyzed the data and wrote the paper. All authors read and approved the final manuscript.
